# LRRC8-Mediated Glutamate Release from Astrocytes Is Not Increased During the Initiation of Experimental Temporal Lobe Epilepsy

**DOI:** 10.3390/ijms27031589

**Published:** 2026-02-05

**Authors:** Kamyab Sarmadi, Linda Gaspar, Peter Bedner, Lukas Henning, Christian Henneberger, Ronald Jabs, Thomas J. Jentsch, Christian Steinhäuser, Gerald Seifert

**Affiliations:** 1 Institute of Cellular Neurosciences I, University Hospital Bonn, University of Bonn, 53127 Bonn, Germany; kamyabsarmadi@gmail.com (K.S.); linda.gaspar@gmx.de (L.G.); peter.bedner@ukbonn.de (P.B.); lukas.henning@ukbonn.de (L.H.); christian.henneberger@uni-bonn.de (C.H.); ronald.jabs@ukbonn.de (R.J.); 2 German Center for Neurodegenerative Diseases (DZNE), 53127 Bonn, Germany; 3 Leibniz-Forschungsinstitut für Molekulare Pharmakologie (FMP), 13125 Berlin, Germany; jentsch@fmp-berlin.de; 4 NeuroCure Cluster of Excellence, Charité Universitätsmedizin Berlin, 10117 Berlin, Germany

**Keywords:** VRAC, VSOR, SWELL1, chloride channel, astrocyte, epilepsy

## Abstract

LRRC8 channels are volume-regulated anion channels (VRACs) activated by cellular swelling, which mediate regulatory volume decrease in many cell types. Recently, it has been shown that these channels contribute to the release of glutamate from astrocytes. Since enhanced extracellular glutamate concentrations produce hyperexcitability, and microdialysis revealed elevated levels of the transmitter in the brains of epileptic patients, we asked whether astroglial glutamate release through LRRC8/VRACs might contribute to the initiation of experimental temporal lobe epilepsy (TLE). Patch clamp, pharmacological, and single-cell transcript analyses were performed in the hippocampus of controls and mice with inducible deletion of LRRC8a in astrocytes. In addition, these mice were exposed to our unilateral intracortical kainate model of TLE. Tonic currents were recorded from CA1 pyramidal neurons as a measure of glutamate release. Our data show that neither expression of LRRC8a nor the amplitude of tonic currents was altered 4 h after status epilepticus-induced TLE. These findings do not suggest that increased astroglial glutamate release through LRRC8 channels contributes to the initiation of experimental TLE.

## 1. Introduction

Extracellular K^+^ accumulation, acidification, and glutamate accumulation have been proposed to cause astrocytic swelling under pathophysiological conditions, such as ischemia and traumatic brain injury [1]. Cellular swelling activates volume-regulated anion channels (VRACs), which, together with parallel efflux of K^+^ and water, mediate regulatory volume decrease in various cell types [2,3,4,5,6]. Importantly, VRACs not only conduct inorganic anions, but also various organic molecules, including neurotransmitters and ATP, that may exacerbate excitotoxicity and cellular stress [2,3,4,7,8,9,10]. VRACs are composed of the Leucine Rich Repeat Containing 8a (LRRC8a) subunit (also called SWELL1) [3,4] and any of the other members of the LRRC8 family (LRRC8b-LRR8e) with which it forms heteromers [3,7,11,12]. LRRC8a is essential for channel function and plasma membrane localization of LRRC8 heteromers [3]. It is ubiquitously expressed, including in neurons and astrocytes. LRRC8 channels are activated Ca^2+^-independently under hypoosmolar conditions and by several signalling pathways, including those triggered by ATP receptors. They can be inhibited by unspecific Cl^−^ channel inhibitors 4-(2-Butyl-6,7-dichloro-2-cyclopentyl-1-oxo-2,3-dihydro-1H-inden-5-yloxy)butanoic acid (DCPIB) and 5-Nitro-2-(3-phenylpropylamino)benzoic Acid (NPPB). LRRC8 proteins are structurally related to pannexins [13], possess 4 transmembrane helices, and form hexamers [11,13,14,15].

Astrocytes serve homeostatic functions, such as uptake of K^+^ ions and the neurotransmitters glutamate and GABA, energetic supply of nerve cells with lactate, and the synthesis and release of neurotransmitters, the so-called gliotransmitters, e.g., glutamate, D-Serine, or ATP [16,17,18,19,20]. Through gliotransmitter-mediated actions, astrocytes can interact with neuronal circuits [21]. The mechanisms of gliotransmitter release have been controversially discussed [22,23]. Apparently, only a subpopulation of astrocytes in the adult brain is able to release glutamate through regulated exocytosis from presynaptic vesicle-like structures in a Ca^2+^-dependent manner [24,25]. Besides exocytosis, the release of neurotransmitters through anion channels came into the focus of glial research. From today’s perspective, the most convincing evidence for glutamate release through astrocytic anion channels exists for LRRC8 channels [10,26,27,28]. In mice with mGFAP-driven conditional knockout of LRRC8a, the frequency of miniature EPSCs, LTP, and the probability of presynaptic glutamate release are reduced, indicative of reduced glutamate release from astrocytes [29].

In temporal lobe epilepsy (TLE), a frequent form of adult focal epilepsies, transmitter and electrolyte homeostasis are disrupted and critically contribute to the pathogenesis [30]. We have established a mouse model of human TLE based on unilateral intracortical kainate injection, which reproduces key changes typical of the chronic stage of the disease, including the generation of spontaneous generalized seizures and unilateral hippocampal alterations such as neuronal loss, complete loss of glial gap junction coupling in the CA1 region of the hippocampus, astrogliosis, and granule cell dispersion [31,32]. In this model, release of TNFα, astroglial coupling, and K^+^ buffering are disturbed as early as 4 h after induction of status epilepticus (SE), i.e., before the onset of neuronal changes, suggesting that these glial dysfunctions trigger or initiate TLE [31,33]. However, increased glutamate release from astrocytes may also cause neuronal synchronization and epileptogenesis, and elevated extracellular glutamate levels have been detected in TLE patients [34,35]. Here, we asked whether, in addition to the alterations reported above, SE-induced glutamate release from astrocytes can contribute to the initiation of TLE. To avoid additional disruption in neurons, as, for example, seen after mGFAP-driven conditional knockout, we used a mouse line allowing for tamoxifen-induced deletion of LRRC8a specifically in astrocytes of the adult brain. We report that in astrocytes of acute hippocampal slices, hypoosmolar solutions evoked currents through voltage-regulated anion channels (VRACs), which were sensitive to the VRAC blocker NPPB. Single-cell transcript analysis identified LRCC8a in almost all astrocytes analyzed. By recording tonic NMDA receptor currents from CA1 pyramidal neurons, we identified VRAC-dependent release of glutamate from astrocytes, which was inhibited by the VRAC blocker DCPIB and was absent in mice with astrocyte-specific deletion of LRRC8a. In our mouse model of TLE, no changes in LRRC8-mediated glutamate release were observed in control mice during the first 4 h after SE, suggesting that this mechanism does not contribute to the very early events of TLE initiation.

## 2. Results

### 2.1. Activation of Swelling-Induced Membrane Currents in Hippocampal Astrocytes

**Figure 1:**
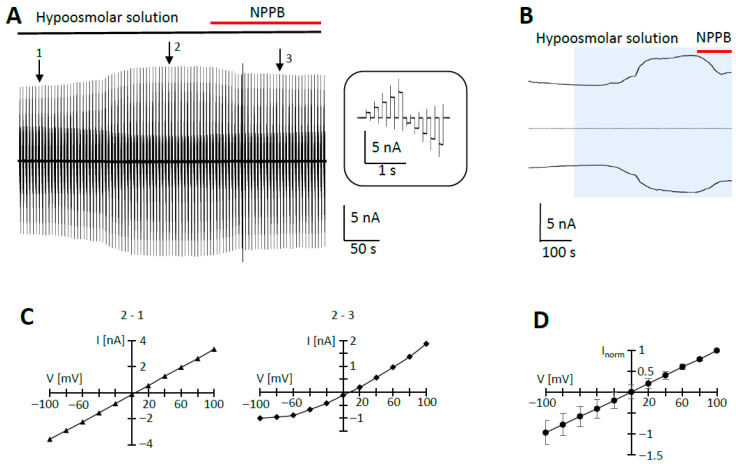
Swelling-induced currents in hippocampal astrocytes in situ (hGFAP-EGFP mice). (**A**) In the presence of K^+^-free intra- and extracellular solutions and the gap junction blocker MFA (100 µM), a hypoosmolar bath solution (220 mOsm) was applied (black horizontal line). During application, the cell membrane was repetitively de- and hyperpolarized from −100 to +100 mV for 100 ms (20 mV increments, every 4.5 s, holding potential 0 mV). A representative current response is shown in the inset. The anion channel blocker NPPB (100 µM) was applied as indicated by the red horizontal line. (**B**) Membrane currents induced by hypoosmolar solution (blue box) of the same cell (at ±100 mV) and the effect of NPPB (red line). (**C**) Respective I/V relation of swelling-induced currents after subtraction of membrane currents, as indicated by the arrows in (**A**). NPPB-sensitive responses are shown on the right. (**D**) Averaged I/V relationship of swelling-induced currents (mean ± SD; n = 10). For each cell, data were normalized to the maximum outward current.

To isolate swelling-induced Cl^−^ currents in hippocampal astrocytes, K^+^-free intra- and extracellular solutions were used, and gap junctions were blocked with meclofenamic acid (MFA, 100 µM). Under these conditions, wash-in of hypoosmolar bath solution slowly (3–5 min) induced large membrane currents (Figure 1A,B). Under iso-stoichiometric Cl^−^ concentrations, swelling-induced currents reversed at −3.5 ± 17.2 mV (number of cells (n) = 10; number of animals (N) = 8). The mean I/V relation of swelling-induced currents was almost linear (Figure 1C,D). At membrane potentials of +100 mV and −100 mV, currents increased to 151 ± 33% and 151 ± 35% compared to the prior control conditions (n = 10). The currents were inhibited by NPPB, a VRAC blocker (Figure 1A–C). Our data show that in hippocampal astrocytes, VRACs open under hypoosmotic conditions.

### 2.2. Molecular Identity of Anion Channels Expressed by Astrocytes in the Hippocampus

**Figure 2:**
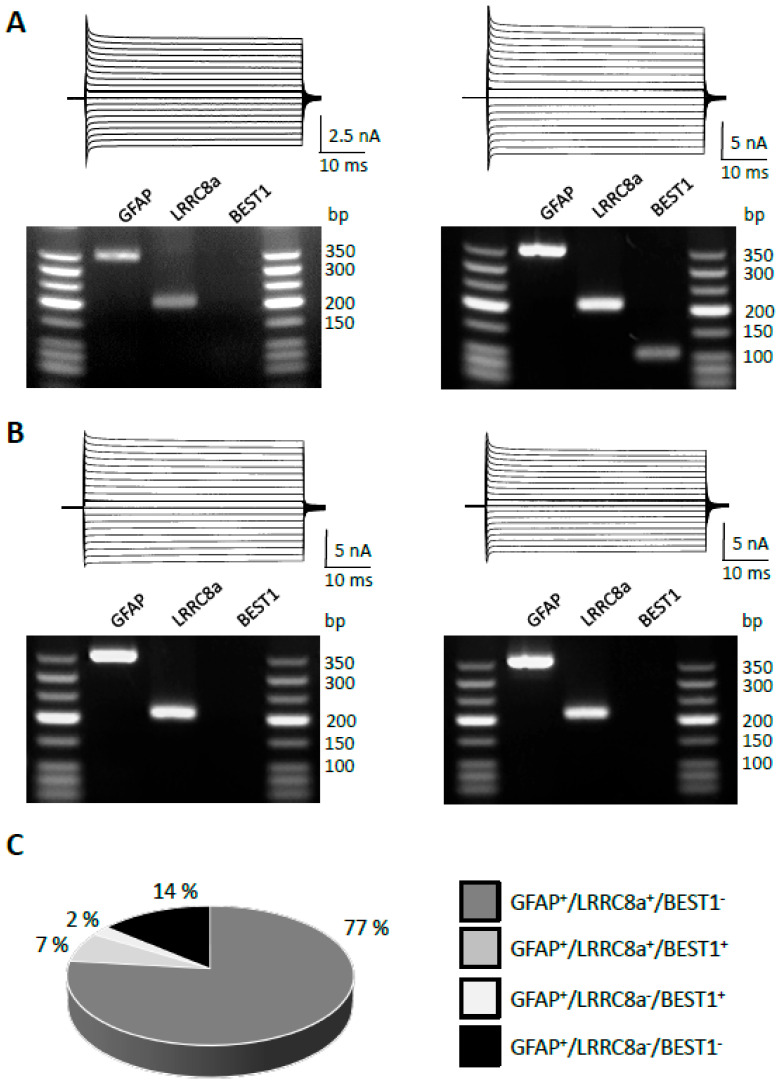
Expression of Cl^−^ channels in single hippocampal astrocytes. (**A**) Cells were de- and hyperpolarized from −160 to +20 mV for 50 ms (10 mV increments, holding potential −80 mV; hGFAP-EGFP mice). After recording, the cell content was harvested, and RT-PCR was performed. VRACs were co-expressed with other Cl^−^ channels, as shown in the agarose gels below the corresponding current traces. The expected product lengths were 208 bp for LRRC8a and 106 bp for Best1. GFAP (350 bp) served as an astrocyte-specific housekeeping gene. (**B**) Same as in (**A**), but astrocytes were harvested from hippocampi of C57BL/6 mice. (**C**) The majority of GFAP mRNA-positive cells (i.e., astrocytes) co-expressed LRRC8a (77% + 7%). One cell expressed GFAP and BEST1 (2%), while 14% of the astrocytes analyzed lacked LRRC8a and BEST1 gene transcripts. The total number of cells was n = 43 (N = 11).

Astrocytes express several types of anion channels, which may regulate cellular volume and neurotransmitter release. Besides LRRC8 channels, the Ca^2+^-activated Cl^−^ channel Best1 might have pharmacological properties similar to LRRC8 and release glial GABA and glutamate [36,37]. To identify the anion channels expressed by astrocytes in the hippocampus, we applied single-cell RT-PCR subsequent to electrophysiological characterization (Figure 2A,B). GFAP was used as a housekeeping gene. A vast majority of GFAP mRNA-positive astrocytes in the CA1 stratum radiatum region of the hippocampus expressed transcripts encoding LRRC8a (84%), while Best1 was rarely found (9%, n = 43; Figure 2C).

### 2.3. Tonic NMDA Receptor Currents of CA1 Pyramidal Neurons Are Sensitive to Anion Channel Blockers

**Figure 3:**
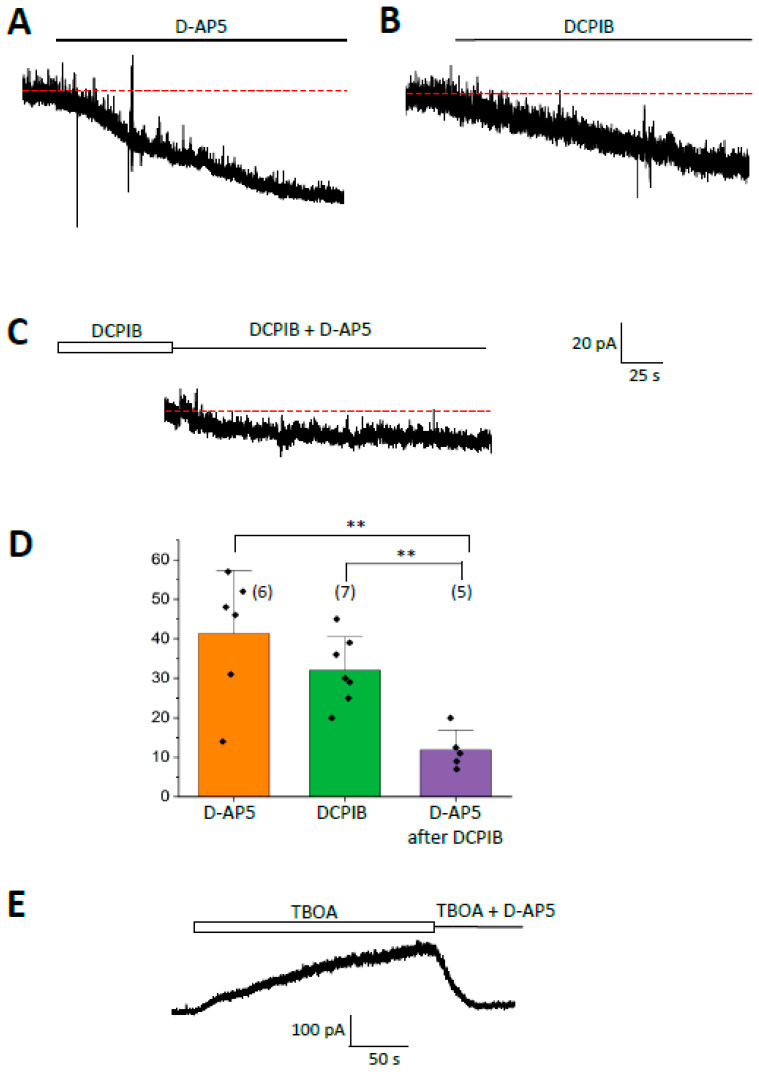
Activation of tonic NMDA receptor currents and their sensitivity to NMDA receptor and VRAC inhibitors (C57BL/6 mice). (**A**) In the presence of blockers of AMPA receptors, GABA_A_ receptors, Na^+^, and K^+^ channels, CA1 pyramidal neurons were held at +40 mV. Bath application of D-AP5 (50 µM) blocked a current of about 52 pA. (**B**) In another cell, bath application of DCPIB (25 µM) reduced the amplitude of tonic currents by 36 pA. (**C**) In the same cell, adding D-AP5 to the DCPIB-containing solution blocked only a small residual current of about 13 pA. (**D**) Summary of blocking effects on tonic NMDA receptor currents by D-AP5, DCPIB, and D-AP5 after prior DCPIB application. (**E**) Application of TBOA (100 µM) increased the amplitude of tonic NMDA-receptor currents, which were blocked by subsequent application of D-AP5 (25 µM). Bar graphs: Mean ± SD; number of cells is given in brackets. Red dashed lines represent holding current before drug application. Significant differences are indicated by asterisks (two-sample *t*-test). ** (*p* < 0.01).

Recently, it was reported that even under normosmolar conditions, LRRC8a channels in hippocampal astrocytes mediate tonic glutamate release, activating neuronal NMDA receptors [29]. We confirmed these findings with whole-cell patch clamp recordings from CA1 pyramidal neurons (holding potential +40 mV). NMDA receptor currents (outwardly directed at +40 mV) were isolated with a Cs^+^-based pipette solution in ACSF containing NBQX (10 µM), picrotoxin (100 µM), and TTX (0.5 µM) to block AMPA and GABA receptors as well as Na^+^ and K^+^ currents. The isolated tonic outward current (41.3 ± 16.0 pA; n = 6, N = 6) was reduced by bath application of D-2-amino-5-phosphonopentanoate (D-AP5, 50 µM), an NMDA receptor antagonist [38] (Figure 3A,D). To test whether the glutamate responsible for this NMDA receptor current component was released through VRACs, DCPIB (25 µM) was added to the bath solution, and tonic currents were recorded from neurons at +40 mV. DCPIB inhibited a current fraction similar to that blocked by D-AP5 (32.0 ± 8.6 pA; n = 7, N = 6; *p* = 0.24) (Figure 3B,D). In the presence of DCPIB, application of D-AP5 led to a further reduction in the tonic current (by 11.9 ± 5.0 pA, n = 5, N = 5; *p* = 0.005 when comparing to sole D-AP5 application) (Figure 3C,D). Thus, tonic NMDA receptor currents of hippocampal CA1 neurons were effectively inhibited by anion channel blockade.

Tonic NMDA receptor currents are regulated by both glutamate release and uptake [39,40]. In our hands, application of the glutamate transporter antagonist TBOA (100 µM) led to a strong increase in tonic NMDA receptor currents (up to 376 ± 225 pA; n = 7, N = 6), which was almost completely (by 96 ± 9%) blocked by D-AP5 (n = 3, N = 3; Figure 3E). Our data show that tonic NMDA receptor currents are sensitive to anion channel blockers and increased by inhibition of glutamate uptake. The residual D-AP5-sensitive currents remaining in the presence of DCPIB might suggest that other sources of extracellular glutamate exist besides VRAC anion channel-mediated release.

### 2.4. Tonic NMDA Receptor Currents of CA1 Pyramidal Neurons Are Reduced in LRRC8a Knockout Mice

**Figure 4:**
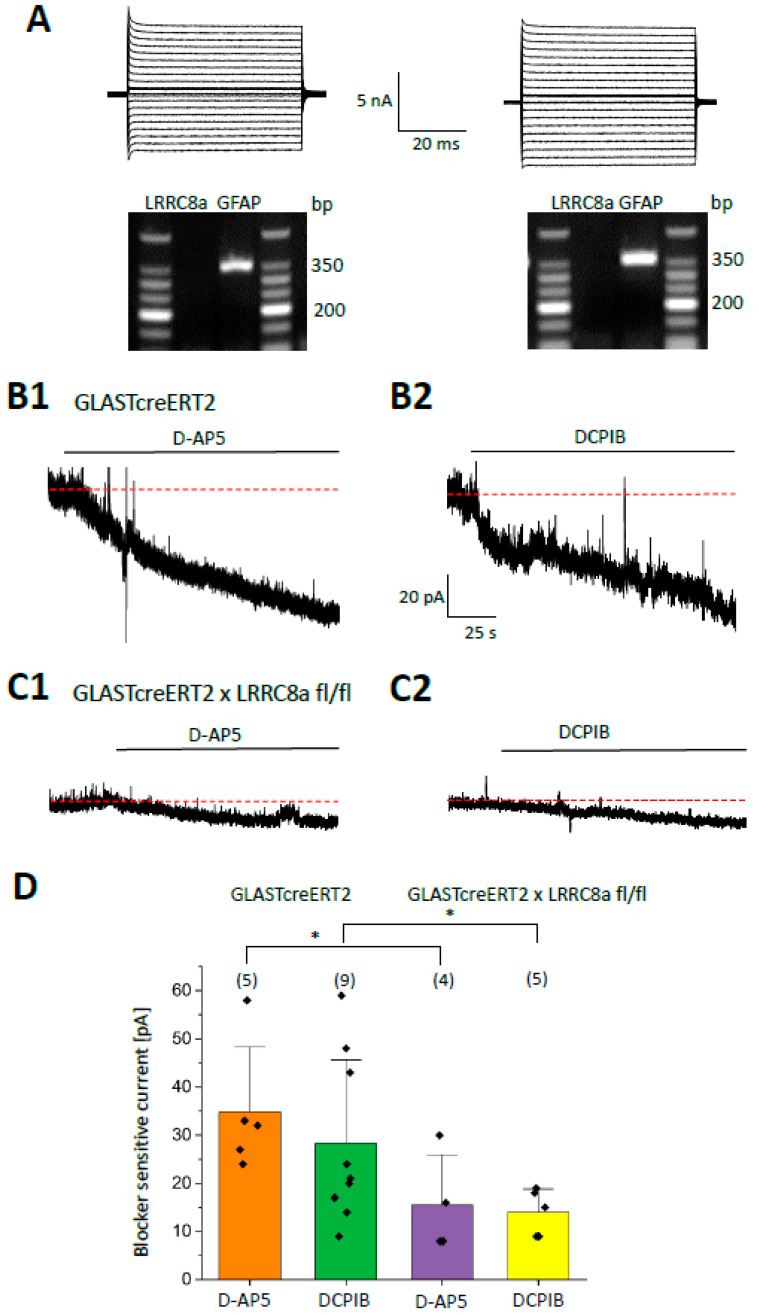
Tonic NMDA receptor currents are reduced after deletion of LRRC8a. (**A**) Two representative astrocytes from tamoxifen-injected LRRC8a KO mice were electrophysiologically characterized in situ as described in the legend of Figure 2 (upper panels). Subsequently, the cell content was harvested, and RT-PCR was performed. Both cells were devoid of LRRC8a (expected product length 208 bp), while the astrocyte-specific housekeeping gene GFAP (350 bp) was present. (**B1**,**B2**) In the presence of blockers of AMPA receptors, GABA_A_ receptors, Na^+^ and K^+^ channels, CA1 pyramidal neurons were clamped at +40 mV (GLASTcreERT2 mice). Bath application of D-AP5 (50 µM) and DCPIB (25 µM) blocked currents by 58 and 48 pA, respectively. (**C1**,**C2**) In LRRC8a KO mice, the same protocols reduced the tonic currents only by about 8 pA and 9 pA, respectively. (**D**) Summary of the blocking effects on tonic NMDA receptor current by D-AP5 and DCPIB in the two mouse strains. Mean ± SD, number of cells in brackets. Red dashed lines represent holding current before drug application. Significant differences are indicated by asterisks (two-sample *t*-test). * (*p* < 0.1).

To test whether VRACs mediate glial glutamate release and activation of neuronal NMDA receptors, mice with inducible and conditional LRRC8A KO (GLASTcreERT2 x LRRC8a fl/fl) were used. In these mice, VRACs are specifically deleted in postnatal astrocytes after tamoxifen injection. Recombination efficacy was tested by harvesting CA1 astrocytes in situ, 3 weeks after tamoxifen injection (p53–p59) and performing single-cell RT-PCR. Under these conditions, the majority (69%) of GFAP-expressing astrocytes were devoid of LRRC8a mRNA (Figure 4A) (n = 16, N = 4). All experiments in LRRC8a KO mice were performed 3 weeks after tamoxifen injection; GLASTcreERT2 mice served as controls. In control animals, D-AP5 and DCPIB inhibited neuronal tonic outward currents recorded at +40 mV by 34.8 ± 13.5 pA (n = 5, N = 2) and 28.3 ± 17.3 pA (n = 9, N = 4), respectively (Figure 4B1,B2,D). In tamoxifen-treated LRRC8a KO mice, both blockers were less efficient (D-AP5: inhibition by 15.5 ± 10.4 pA, n = 4, N = 3, *p* < 0.05 compared to its effect in controls; DCPIB: by 14.0 ± 4.8 pA, n = 5, N = 3, *p* < 0.05; Figure 4C1,C2,D). The residual DCPIB-sensitive current observed in LRRC8a KO mice might indicate glutamate release through LRRC8a from non-recombined astrocytes and from neurons. In conclusion, tonic NMDA receptor currents were reduced in astrocyte-specific LRRC8a KO mice, suggesting that astrocytes can release glutamate through VRACs.

### 2.5. LRRC8a-Mediated Responses Remain Unchanged After Status Epilepticus in a Mouse Model of TLE

**Figure 5:**
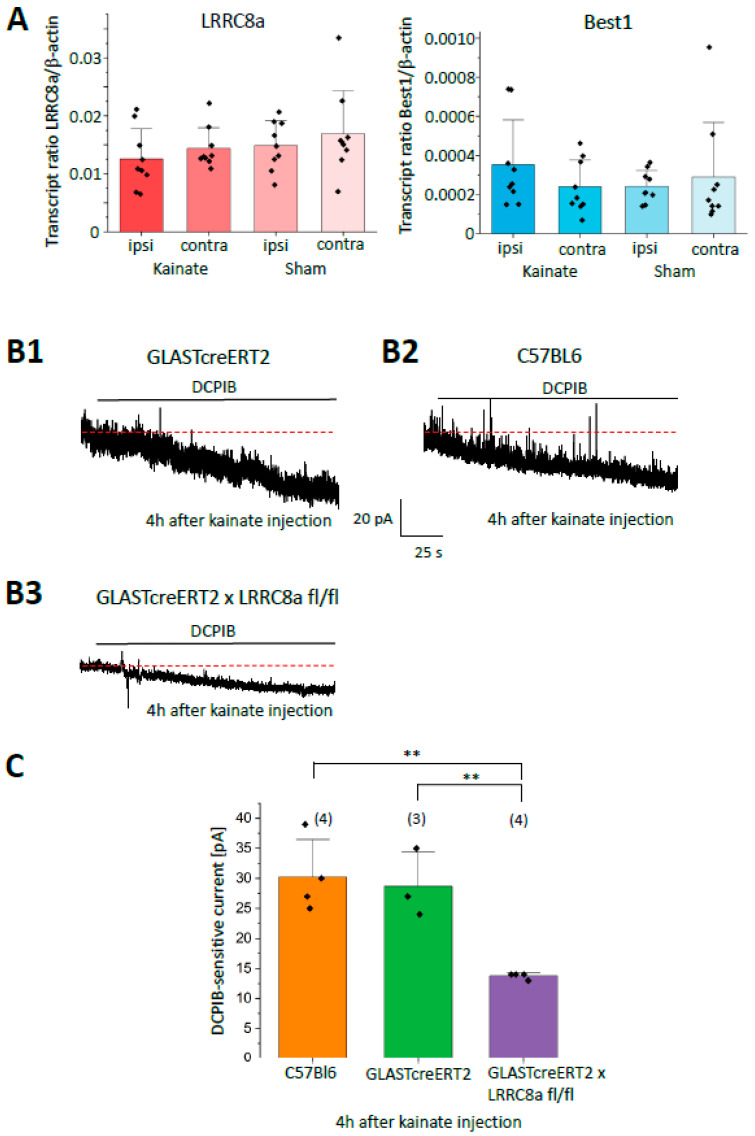
Transcript analyses and tonic NMDA receptor currents after status epilepticus. (**A**) RT-qPCR was performed with mRNA extracted from hippocampi, 4 h after unilateral intracortical injection of kainate and NaCl (sham control) (C57BL/6 mice). Expression ratios relative to β-actin were determined for LRRC8a and Best1 (cf. Equation (1)). Mean ± SD obtained from 9 slices/3 mice, each. (**B1**–**B3**) Membrane currents and sensitivity to DCPIB (25 µM) of CA1 pyramidal neurons from mouse lines as indicated, 4 h after kainate injection (for recording protocols, see legend to Figure 3). (**C**) Summary of the blocking effects of DCPIB on tonic NMDA receptor current, analyzed 4 h after kainate injection. Mean ± SD, number of cells is given in brackets. Red dashed lines represent holding current before drug application. Significant differences are indicated by asterisks (*p* < 0.01; one-way ANOVA and Tukey post hoc test). ** (*p* < 0.01).

To investigate whether astroglial glutamate release through LRRC8a contributes to the initiation of epilepsy, we employed the unilateral intracortical kainate model of chronic TLE, which recapitulates key features of the human disease [31,32]. First, we used semi-quantitative RT-PCR to test whether kainate injection affects mRNA expression of LRRC8a or BEST1 in the hippocampus of C57BL/6 mice. mRNA was isolated ipsilaterally from tissue slices underneath the injection site and from corresponding contralateral slices. NaCl injections served as controls. None of the injections led to changes in the expression of LRRC8a or Best1, neither ipsilaterally nor on the contralateral side (Figure 5A). It is noteworthy that hippocampal LRRC8a expression levels exceeded those of Best1 by about fiftyfold.

To look for potential functional changes, we examined tonic outward currents in the hippocampus of C57BL/6, GLASTcreERT2, and tamoxifen-injected GLASTcreERT2 x LRRC8a KO mice, 4 h after kainate injection. Currents were recorded at +40 mV from CA1 pyramidal neurons in ipsilateral slices, about 100–200 µm underneath the injection site. After kainate, DCPIB inhibited the tonic current in C57BL/6 and GLASTcreERT2 mice by 30.3 ± 6.2 pA (n = 4, N = 3) and 28.7 ± 5.7 pA (n = 3, N = 2), respectively (Figure 5(B1–B3),C). In kainate-injected LRRC8a KO mice, DCPIB reduced the currents by 13.7 ± 0.5 pA (n = 4; N = 3; *p* < 0.05 as compared to C57BL/6 and GLASTcreERT2 control). This block was similar to the one observed in the respective mouse lines without kainate treatment (cf. Figure 3D and Figure 4C; *p* < 0.01 and *p* < 0.05 for C57Bl/6 and GLASTcreERT2, respectively). Together, our data do not support a contribution of LRRC8a to the initiation of epilepsy in our TLE model.

## 3. Discussion

Being part of tripartite synapses, astrocytes release gliotransmitters and thereby actively contribute to information processing in the brain. In addition to Ca^2+^-dependent exocytosis [24,25], increasing evidence suggests that astrocytes are also capable of ion channel-mediated release of gliotransmitters, particularly through VRACs [9,41,42]. Previous work has shown that glutamate release is reduced in mice with mGFAP-driven conditional knockout of LRRC8a, resulting in less frequent miniature EPSCs, reduced LTP, and lower probability of presynaptic glutamate release [29]. Conditional LRRC8a knockout using the Nestin-Cre strategy led to complete loss of LRCC8a in neurons and glial cells, generation of spontaneous seizures, and subsequent premature death [43]. Similar to findings of another study, complete LRRC8a deletion reduced stroke damage after middle cerebral artery occlusion (MCAO) [44]. In contrast, applying the same MCAO stroke model to mice with astrocyte-specific, inducible LRRC8a knockout under control of Aldh1l1-driven Cre-activity produced no protection but resulted in increased lesion size. It was concluded that LRRC8a contributes to ischemic brain injury through mechanisms other than VRAC-mediated glutamate release [45]. Obviously, the contribution of astroglial LRRC8a channels and glutamate release to brain pathology needs further clarification.

In the present study, we investigated whether, in addition to the reported impairment of glial gap junction coupling and K^+^ buffering [31], SE-induced glutamate release through astroglial LRRC8a is increased and might contribute to the initiation of experimental TLE. Indeed, increased glutamate release causes hyperexcitability and epileptogenesis, and the extracellular glutamate concentration is increased in the brains of TLE patients [34,35]. To avoid recombination in neurons and ensure high recombination efficiency selectively in astrocytes of the adult brain, we used a mouse line with tamoxifen-inducible, GLAST-controlled LRRC8a deletion (recombination efficiency > 98%; astrocyte specificity 90%; [46]). In our study, neither expression of LRRC8a nor the amplitude of tonic currents was altered 4 h after SE-induced TLE, making it unlikely that enhanced astroglial glutamate release through LRRC8a contributes to the initiation of TLE.

Although VRAC currents were known for decades, their molecular identity as LRRC8 heteromers was only established in 2014 in various cell types [3,4,6], including astrocytes [10,42,47]. As expected, in control mice, application of hypoosmolar solutions under iso-stoichiometric Cl^−^ concentrations provoked membrane currents in hippocampal astrocytes, which, however, could only be seen after reducing currents through K^+^ channels and gap junctions. Due to incomplete suppression of the latter, the resulting I/V relations were rather linear. These currents were in part sensitive to NPPB. The blocker-sensitive currents showed outward rectification similar to LRRC8 channels. Best1, a Ca^2+^-activated anion channel proposed to be involved in GABA and glutamate release from astrocytes [36,48,49,50] is expressed at lower levels in the hippocampus. In line with these previous findings, our single-cell transcript analyses confirmed expression of LRRC8a, but not Best1, in the vast majority of astrocytes investigated.

Even under physiological, i.e., isoosmolar conditions, VRACs may open [5,10] and account for so-called leaked glutamate, which may activate tonic NMDA receptor currents in neurons [38,51] or presynaptic mGluR receptors [29]. To test if hippocampal astrocytes release glutamate under physiological conditions, we monitored tonic currents in CA1 pyramidal neurons. D-AP5 inhibited these tonic currents in control mice, suggesting that they were mediated by NMDA receptors. DCPIB, an inhibitor of VRACs and other anion channels [52], also inhibited the tonic currents. Adding D-AP5 to the DCPIB-containing solution in slices from control mice still inhibited a small current. In LRRC8a knockout mice, tonic currents were reduced, but residual D-AP5-sensitive responses still remained. Thus, astrocytic LRRC8a-based VRACs are only partly responsible for tonic outward currents in neurons.

Since extracellular ATP activates VRACs [10] and ATP is released from neurons or astrocytes during seizure activity [53,54,55], we asked whether enhanced VRAC-mediated glutamate release might contribute to the initiation of epilepsy using our unilateral intracortical kainate model of TLE [31,33]. We did not observe an increase in DCPIB- or D-AP5-sensitive currents during the first hours after kainate-induced SE. However, enhanced VRAC-mediated glutamate release might contribute to later stages of epileptogenesis because increased LRRC8a protein was observed in another TLE model from the 7th day after systemic kainate injection [56]. Whether glutamate is released through other astrocytic pathways, e.g., connexin hemichannels [57,58] might contribute to the initiation or maintenance of TLE remains to be investigated.

In conclusion, our findings are not in favour of the idea that LRRC8-mediated glutamate from astrocytes contributes to the initiation of TLE. Additional experiments are needed to clarify whether a reduction in glial glutamate release through inhibition of VRACs attenuates epileptogenesis at later stages, as previously suggested [27].

## 4. Materials and Methods

### 4.1. Animals

Experiments were performed with C57BL/6 (Charles River, Sulzfeld, Germany, or bred in-house), hGFAP-EGFP [59,60], GLASTcreERT2 [61], and GLASTcreERT2 x LRRC8a fl/fl [62] mice (both sexes, aged postnatal days (p) p58–91). To induce selective knockout of *LRRC8A* in astrocytes, mice were intraperitoneally injected with tamoxifen (1.5 mg once per day for 4 consecutive days, at least 3 weeks before experiments). On the day of the first injection, the animals were at least 25 days old. Maintenance and handling of animals were performed according to EU and local governmental regulations. Experiments were approved by the North Rhine-Westphalia State Agency for Nature, Environment, and Consumer Protection (approval number 81-02.04.2020.A420, approval date 23 February 2021). All measures were taken to minimize the number of animals. Mice were kept under standard housing conditions (12 h/12 h light-dark cycle) with food and water provided ad libitum.

### 4.2. Slice Preparation

Mice were anesthetized with isoflurane (Piramal Critical Care, Voorschoten, The Netherlands) and sacrificed. Brains were dissected, and tissue was cut into 200 µm thick frontal slices using a vibratome (VT1200S, Leica Biosystems, Nussloch, Germany) in ice-cold preparation solution (in mM: 87 NaCl, 2.5 KCl, 1.25 NaH_2_PO_4_, 25 NaHCO_3_, 7 MgCl_2_, 0.5 CaCl_2_, 25 glucose, 61 sucrose; 335 mOsm) gassed with carbogen (95% O_2_/5% CO_2_). Subsequently, the slices were transferred into preparation solution for 15 min (35 °C, gassed with carbogen) and then incubated in artificial cerebrospinal fluid (ACSF, in mM: 126 NaCl, 3 KCl, 1.25 NaH_2_PO_4_, 2 MgSO_4_, 2 CaCl_2_, 10 glucose, 26 NaHCO_3_, gassed with carbogen at room temperature; 305–315 mOsm, pH 7.35). To aid identification of astrocytes in the tissue, ACSF was supplemented with SR101 (1 μM, Merck, Darmstadt, Germany; incubation 20 min, 35 °C) [63,64]. Subsequently, brain slices were stored in ACSF at room temperature.

### 4.3. Electrophysiological Recordings

Slices were transferred to a recording chamber and continuously perfused with carbogen-gassed ACSF. Cells were visualized using an upright microscope equipped with infrared differential interference contrast using 60× water immersion objectives (Eclipse E600FN, Nikon Europe, Amstelveen, The Netherlands). Patch clamp pipettes were fabricated from borosilicate glass capillaries (Science Products, Hofheim, Germany) by a horizontal puller (DMZ Zeitz-Puller, Zeitz, Martinsried, Germany) and had a resistance of 3–6 MΩ. The pipette solution for recording from neurons was composed of (in mM): 100 Cs-gluconate, 4 NaCl, 10 TEA-Cl, 1 MgCl_2_, 2 Na_2_-ATP, 0.3 GTP, 5 phosphocreatine,10 2-[4-(2-hydroxyethyl)-piperazine-1-yl] ethanesulfonic acid (HEPES), 10 1,2-bis(2-aminophenoxy)-N,N,N,N′-tetraacetic acid (BAPTA; 290 mOsm, pH 7.28). For cell harvesting, a KCl-based intracellular solution was used, containing (in mM): 130 KCl, 10 HEPES, 5 BAPTA, 2 MgCl_2_, 0.5 CaCl_2_; 290 mOsm, pH 7.28. Recordings were performed in the whole cell configuration, liquid junction potential (−10 mV) was corrected online. Recordings were performed at room temperature. Chemicals were purchased from AppliChem GmbH (Darmstadt, Germany), Fluka, and Carl Roth (Karlsruhe, Germany). To isolate tonic NMDA receptor currents, ACSF was supplemented with (in µM): 10 NBQX, 100 picrotoxin (PTX), 0.5 TTX, 10 D-serine, and the holding potential was −60 mV. Neurons were depolarized to +40mV [38]. When recordings were stable for at least 5 min, DCPIB (25 µM) and/or D-AP5 (50 µM) were added to the bath to block LRRC8a and NMDA receptors. An EPC9 amplifier (Heka, Lambrecht, Germany) was used; signals were filtered at 3 or 10 kHz and sampled at 6 or 20 kHz. TIDA 5.25 software (HEKA) and Igor Pro 6.37 software (WaveMetrics, Lake Oswego, OR, USA) were used for data acquisition and analysis.

### 4.4. Evaluation of Swelling-Induced Currents of Astrocytes in the Hippocampal CA1 Region

To investigate swelling-induced Cl^−^ currents in astrocytes, whole-cell patch clamp recordings were performed. Astrocytes were identified by their SR101 fluorescence [64], characteristic shape, and membrane current pattern. The holding potential was −80 mV, and only cells with an input resistance <20 MΩ were considered. After baseline recording was stable for at least 5 min, the bath was switched to the following isoosmolar solution (in mM): 90 NMDG-Cl, 2 KCl, 1 MgCl_2_, 2 CaCl_2_, 10 HEPES, 10 glucose, and 100 mannitol (pH adjusted to pH 7.4 with NMDG; osmolality was 310 mOsm). After another 5 min, to induce swelling, a hypotonic bath solution lacking mannitol was applied (220 mOsm; cf. [29]). To block VRACs’ opening under hypotonic conditions, DCPIB (25 µM) or NPPB (100 µM) was applied.

### 4.5. Unilateral Intracortical Kainate Injection

We employed the mouse model for TLE with hippocampal sclerosis (TLE-HS) previously described [31,32]. Briefly, mice were anesthetized via i.p. injection of a mixture of medetomidine (Cepetor, CP-Pharma, Burgdorf, Germany; 0.3 mg/kg) and ketamine (Ketamidor, WDT, Garbsen, Germany; 40 mg/kg), and placed in a stereotaxic frame equipped with a manual microinjection unit (David Kopf Instruments, Tujunga, CA, USA). A total volume of 70 nL of a 20 mM kainate solution (Tocris, Bristol, UK) dissolved in 0.9% sterile NaCl was injected into the neocortex, directly above the right dorsal hippocampus. Stereotaxic coordinates relative to bregma were: 2.0 mm posterior, 1.5 mm lateral, and 1.7 mm below the skull surface. Sham control mice received injections of 70 nL saline under the same conditions. After injection, the scalp incision was sutured, and anesthesia was reversed by i.p. administration of atipamezole (Antisedan, Orion Pharma, Hamburg, Germany; 300 µg/kg). For postoperative analgesia, mice received carprofen (Rimadyl, Pfizer, Karlsruhe, Germany; 4 mg/kg, i.p.). Mice were then returned to clean cages for 4 h prior to experimental use.

### 4.6. Single-Cell RT-PCR

Single-cell transcript analysis was performed as previously reported [60]. Briefly, after slice recording in standard ACSF, the cell at the tip of the pipette was lifted above the slice and aspirated into the recording pipette under microscopic control. The cell content and ~3 µL of the pipette solution were expelled into a reaction tube containing 3 µL of DEPC-treated water. The reaction tube was frozen and stored at −20 °C until reverse transcription (RT) was performed. The RT mastermix contained RT buffer (Thermo Fisher Scientific, Darmstadt, Germany), dNTPs (4 × 250 µM; Applied Biosystems, Darmstadt, Germany), RNasin™ (20 U; Promega, Mannheim, Germany), random hexamer primers (50 µM; Roche, Mannheim, Germany), and reverse transcriptase (Maxima H Minus, 100 U, Thermo Scientific). The reaction mix was added to the harvested cell content; the final volume was 10 µL, and the reaction mix was incubated at 37 °C for 1 h. A multiplex two-round single-cell PCR was performed with primers for LRRC8a, Best1, and the housekeeping gene GFAP (Table 1). The first PCR was performed after adding PCR buffer, MgCl_2_ (2.5 mM), dNTP (4 × 50 µM), primer (200 nM each), and 5 U Taq polymerase (Invitrogen, Darmstadt, Germany) to the RT product (final volume 50 µL). Thirty-five cycles were performed (denaturation at 94 °C, 25 s; annealing at 51 °C, 2 min for the first 5 cycles, and 45 s for the remaining cycles; extension at 72 °C, 25 s; final elongation at 72 °C, 7 min). An aliquot (2 µL) of the PCR product was used as a template for the second PCR (35 cycles; annealing at 54 °C, first 5 cycles: 2 min, remaining cycles: 45 s) using nested primers (Table 1). The conditions were the same as described for the first round, but Platinum Taq polymerase (2.5 U; Invitrogen) was added. Products were identified with gel electrophoresis. As a positive control, total RNA from the mouse brain was run in parallel. Negative controls were performed using distilled water or bath solution for RT-PCR.

### 4.7. RT-sqPCR

To examine LRRC8a and Best1 expression, reverse transcription-semiquantitative polymerase chain reaction (RT-sqPCR) was performed. Tissue samples of the CA1 region of the hippocampus from hGFAP/EGFP mice (p60) were dissected from 200 μm-thick brain slices adjacent to the injection side (ipsilateral) and the corresponding contralateral side of the slice. Total RNA was isolated with Trizol (Thermo Fisher Scientific), precipitated with isopropanol, washed, and dissolved in 10 μL DEPC-treated water. Genomic DNA was removed by DNaseI treatment, and mRNA was isolated using oligo(dT)25-linked Dynabeads (Invitrogen). Dynabeads/mRNA were suspended in DEPC-treated water (20 μL), frozen, and stored at −20 °C. The reaction mix of the RT reaction contained first-strand buffer, dithiothreitol (10 mM), deoxynucleoside triphosphates (4 × 250 µM; all from Thermo Fisher Scientific), random hexamer primers (50 µM; Roche Diagnostics), RNase inhibitor (40 U; Promega), and SuperScript III Reverse Transcriptase (200 U; Thermo Fisher Scientific). The reaction mix (21 μL) and mRNA (20 μL) were incubated at 37 °C for 1 h. The reaction volume for real-time PCR contained PCR mastermix (Takyon, Eurogentec, Seraing, Belgium), TaqMan gene expression assay, and 1 μL cDNA; final volume was 12.5 μL. The reaction mix without cDNA served as a negative control. Samples were denatured at 95 °C (10 min), followed by 50 cycles of PCR (denaturation at 95 °C for 15 s; primer annealing and extension at 60 °C for 1 min). Fluorescence intensity was read out during each annealing/extension step (CFX384 PCR System, Bio-Rad Laboratories, Feldkirchen, Germany). Relative gene expression was determined by comparing threshold cycle values of the target genes with those of the reference gene β-actin at the same fluorescence emission intensity. The relative quantification of different genes was determined according to the equation:

X_target_/X_b-actin_ = 2^^C^_Tβ-actin_^−C^_Ttarget_^ (1)

where X_target_/X_b-actin_ is the gene ratio, and ^C^_T_ the respective threshold cycle numbers.

### 4.8. Data Analysis

Data were normally distributed as confirmed by the Shapiro–Wilk test. Two-sample Student’s *t*-test with or without Welch’s correction, depending on the variances, was performed. For multigroup analysis, data were tested with one-way ANOVA followed by Tukey-test. Data are given as mean ± SD and represented by bar plots. “N” and “n” refer to the number of mice and cells, respectively. Significant differences were indicated by * (*p* < 0.05), ** (*p* < 0.01), *** (*p* < 0.001), and **** (*p* < 0.0001).

## Figures and Tables

**Table 1:** Primers used for single-cell RT-PCR.

Gene	Primer Sequence	Product Length	Position	GenBank Accession Number
LRRC8a	se 5′-ACCGCAACAAAATCGAGAAAATCC as 5′-AGGCCGCTGCGCTTGAGTAGG	343 bp	2009 2331	NM_177725
LRRC8a (nested)	se 5′-ATTGGCCTCCTGCAGAACCTCCAG as 5′-GCTCCACGGGCAGGCATTCC	208 bp	2110 2298	
Best1	se 5′-GTGGAGGGCAAGGATGAGGAAGG as 5′-CACCCAGGGCACCCAGAATGT	219 bp	340 538	NM_011913.2
Best1 (nested)	se 5′-CGCAGCATCAGCACCTCGGTCTAC as 5′-GGCCCAACTTCTGCAACTGCTTAT	106 bp	421 505	
GFAP	se 5′-GCTCCGCCAAGCCAAGCACGAA as 5′-TCACCATCCCGCATCTCCACAGTC	435 bp	809 1220	NM_010277
GFAP (nested)	se 5′-CGCCAACTGCAGGCCTTGACCT as 5′-TGGGCCTTCTGACACGGATTTGGTG	350 bp	850 1176	

Position 1 is the first nucleotide of the initiation codon. The length of PCR products was indicated as base pairs (bp). ‘se’ and ‘as’ mark sense and antisense primers. All sense and antisense primers are located on different exons, respectively.

## Data Availability

The data that support the findings of this study are available from the corresponding author upon reasonable request.
